# Computing with Neural Synchrony

**DOI:** 10.1371/journal.pcbi.1002561

**Published:** 2012-06-14

**Authors:** Romain Brette

**Affiliations:** 1 Laboratoire Psychologie de la Perception, CNRS and Université Paris Descartes, Sorbonne Paris Cité, Paris, France; 2 Equipe Audition, Département d'Etudes Cognitives, Ecole Normale Supérieure, Paris, France; Indiana University, United States of America

## Abstract

Neurons communicate primarily with spikes, but most theories of neural computation are based on firing rates. Yet, many experimental observations suggest that the temporal coordination of spikes plays a role in sensory processing. Among potential spike-based codes, synchrony appears as a good candidate because neural firing and plasticity are sensitive to fine input correlations. However, it is unclear what role synchrony may play in neural computation, and what functional advantage it may provide. With a theoretical approach, I show that the computational interest of neural synchrony appears when neurons have heterogeneous properties. In this context, the relationship between stimuli and neural synchrony is captured by the concept of *synchrony receptive field*, the set of stimuli which induce synchronous responses in a group of neurons. In a heterogeneous neural population, it appears that synchrony patterns represent structure or sensory invariants in stimuli, which can then be detected by postsynaptic neurons. The required neural circuitry can spontaneously emerge with spike-timing-dependent plasticity. Using examples in different sensory modalities, I show that this allows simple neural circuits to extract relevant information from realistic sensory stimuli, for example to identify a fluctuating odor in the presence of distractors. This theory of synchrony-based computation shows that relative spike timing may indeed have computational relevance, and suggests new types of neural network models for sensory processing with appealing computational properties.

## Author Summary

How does the brain compute? Traditional theories of neural computation describe the operating function of neurons in terms of average firing rates, with the timing of spikes bearing little information. However, numerous studies have shown that spike timing can convey information and that neurons are highly sensitive to synchrony in their inputs. Here I propose a simple spike-based computational framework, based on the idea that stimulus-induced synchrony can be used to extract sensory invariants (for example, the location of a sound source), which is a difficult task for classical neural networks. It relies on the simple remark that a series of repeated coincidences *is* in itself an invariant. Many aspects of perception rely on extracting invariant features, such as the spatial location of a time-varying sound, the identity of an odor with fluctuating intensity, the pitch of a musical note. I demonstrate that simple synchrony-based neuron models can extract these useful features, by using spiking models in several sensory modalities.

## Introduction

Neuronal synchronization is ubiquitous in the nervous system [1], [2]. In the retina, neighboring cells are often synchronized at a fine timescale [3], [4], and relative spike timing carries information about visual stimuli [5]. Visual and somatosensory stimulation also elicits synchronized activity in the thalamus [6]–[8], which impacts target cortical neurons [9]–[12]. In olfaction, fine odor discrimination relies on transient synchronization between specific neurons [13]. In the auditory system, phase locking in brainstem neurons [14] produces fine stimulus-driven correlations in spike timing which are determinant for sound localization [15]. At cellular level, modeling and experimental studies show that correlated inputs are more likely to make neurons fire [16]–[19], and synaptic plasticity mechanisms favor correlated synaptic inputs [20], [21], so that developed neural circuits should be very sensitive to correlations. These findings suggest that neural synchronization is functionally important in early sensory pathways, but it is not clear what it implies in terms of computation.

In many theoretical studies of spiking neural networks, spike timing and neural heterogeneity are treated as noise to be averaged out in the activity of “neural masses” [22]. One theory, reservoir computing, assigns a computational role to neural heterogeneity, that of representing sensory stimuli in a high-dimensional space where decoding is easier [23], but it does not assign a specific role to spike timing or synchrony. Thus, although many authors have advocated the idea that the brain may use precise spike timing to process sensory information [24], [25], there are few general theories of spike-based computation. One such theory postulates that the rank order of spikes carries information [26]. This is supported by experimental evidence in the retina [5], but physiologically decoding this information is not entirely straightforward, as it would involve rather specific circuits of inhibition and excitation. In addition, although it seems to be a metabolically efficient way of processing information, the advantages in terms of computational power are not obvious. On the other hand, synchrony can be easily decoded by neurons, by means of coincidence detection [27], and is compatible with Hebbian learning theories, in which correlated inputs tend to be strengthened [20].

In this article, I focus on synchrony induced by the stimulus (rather than by coupling between neurons [28]–[31]) and I address the two following questions: what does synchrony mean? how and what can neurons compute with synchrony? It appears that neural heterogeneity, which is considerable in the nervous system [32], is the key ingredient that makes synchrony computationally interesting, because synchrony then reveals sensory invariants, which play a central role in psychological theories of perception.

## Results

### Synchrony receptive fields

**Figure 1:**
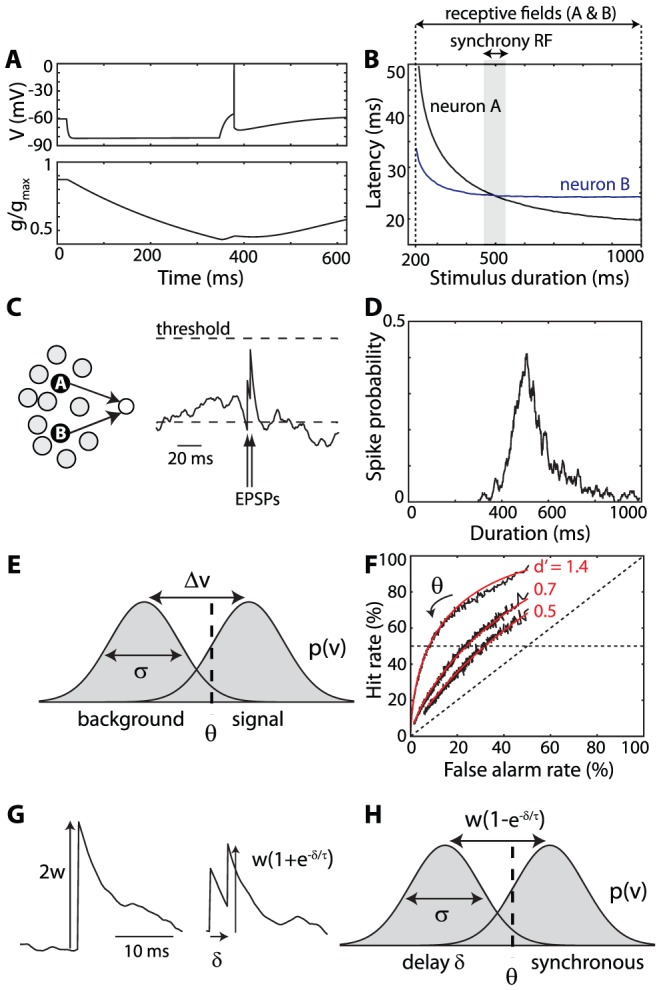
Synchrony receptive field. A, When neuron A is hyperpolarized by an inhibitory input (top), its low-voltage-activated K channels slowly close (bottom), which makes the neuron fire when inhibition is released (neuron models are used in this and other figures). B, Spike latency is negatively correlated with the duration of inhibition (black line). Neuron B has similar properties but different values for the threshold and K channel parameters (blue line). The synchrony receptive field of neurons A and B is the stimulus with duration 500 ms. C, A postsynaptic neuron receives inputs from A and B. D, It is more likely to fire when the stimulus in the synchrony receptive field of A and B. E, Distribution p(v) of the postsynaptic membrane potential when the neuron is not stimulated (left, “background”) and when it receives an input of size Δv (right, “signal”; e.g. neurons A and B shown in panel C fire together). The standard deviation of the distribution is σ. The neuron fires when v is greater than the spike threshold θ. F, Receiver-operation characteristic (ROC) for three levels of noise, obtained by varying the threshold θ (black curves). The hit rate is the probability that the neuron fires within one integration time constant τ when depolarized by Δv, and the false alarm rate is the firing probability without depolarization. The corresponding theoretical curves, with sensitivity index d′ = Δv/σ, are shown in red. G, When a neuron receives two synchronous inputs of size w (PSP peak), the peak potential is 2w plus the background noise (left). When the second input arrives after a delay δ, the peak is 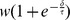 plus the background noise (right). H, Distinguishing between synchronous inputs and delayed inputs corresponds to setting a threshold θ between two distributions separated by 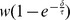.

For synchrony to be computationally useful, it must be stimulus-dependent. To illustrate this idea, let us consider neurons which spike after being hyperpolarized (“rebound spiking”), because of the presence of voltage-activated conductances (Fig. 1; simple neuron models are used in this and all other figures; see Methods for details). Neurons with rebound spiking have been found for example in the superior paraolivary nucleus of the auditory brainstem, a structure involved in encoding the temporal structure of sounds [33]; and in the pyloric network of lobsters, involved in the generation of rhythmic motor patterns [34]. Fig. 1 shows a minimal neuron model with this property (but it is only meant as an illustration). The model includes a slow outward current, modeling K+ channels, which activates at low voltages (half-activation voltage −70 mV). This current prevents the neuron from spontaneously spiking. When the neuron is inhibited for a few hundred ms (Fig. 1A, top), the K+ channels slowly close (the conductance decreases, Fig. 1A, bottom). When inhibition is released, the negative K+ current is smaller than at rest, which makes the neuron spike. The latency of the rebound spike depends on the value of the K+ conductance when inhibition is released, and therefore on the duration of inhibition: if the neuron is inhibited for a shorter duration, K+ channels are still partially open when inhibition is released and the neuron spikes later. If inhibition is very short, the neuron may not spike. Thus, the timing of the rebound spike is negatively correlated with the duration of inhibition. Fig. 1B shows this relationship for two different model neurons A and B, which have the same rebound spiking property but quantitatively different parameter values (spike threshold and time constant of K+ channels).

The receptive field of a neuron can be defined as the set of stimuli which elicit a response in the neuron: in this example, stimuli are inhibitory pulses with duration varying between 0 and 1000 ms, and the receptive fields of neurons A and B are inhibitory pulses lasting more than 200 ms. Therefore, the individual receptive fields of the neurons convey little information about duration. I now define the *synchrony receptive field* (SRF) of two neurons as the set of stimuli which elicit synchronous firing in these two neurons. For neurons A and B in Fig. 1B, the SRF is found at the intersection of the duration-latency curves: the two neurons fire in synchrony when the stimulus lasts about 500 ms. At this point, I make three remarks. First, the SRF reveals information about the stimulus that may not be available from individual receptive fields (here, both neurons fire one spike to all stimuli lasting more than 200 ms). Second, this additional information can only be available when neurons have heterogeneous properties (otherwise, the SRF is the set of all stimuli). Third, the SRF is specific of a pair (possibly group) of neurons: the duration-latency curve of neuron A will generally intersect that of another neuron C at a different point, or may not intersect it at all (and the SRF is empty). Therefore, in a heterogeneous population of neurons, any given stimulus will trigger a specific synchrony pattern. How can this synchrony pattern be decoded?

### Detecting synchrony

Consider a postsynaptic neuron receiving excitatory inputs from neurons A and B (Fig. 1C). The neuron also receives inputs from other sources, which are modeled as background noise. If this neuron is sensitive to coincidences, then it will fire more when the two inputs are synchronous, that is, when the stimulus is in the SRF of A and B. As a result, the firing rate of this neuron will be tuned to the duration of the stimulus, although its inputs are not (Fig. 1D). The model used in Fig. 1D is a simple integrate-and-fire neuron with background noise (time constant τ = 5 ms). As shown in [19] (elaborating on ideas proposed by Abeles [16]), the key ingredient for the neuron to be sensitive to coincidences is that the average background input is subthreshold. In this regime, the neuron is said to be “fluctuation-driven”: it fires to large fluctuations above the mean potential.

This property can be understood in terms of signal detection theory [35]. *In vivo* intracellular recordings show that in many areas, the membrane potential distribution p(v) peaks well below threshold, indicating that neurons are indeed fluctuation-driven (e.g. in auditory cortex [36], visual cortex [37], barrel cortex [38], frontal cortex [39]). This distribution is represented in Fig. 1E (“background”), which we consider as noise with standard deviation σ. When coincident spikes depolarize the neuron by an amount Δv ( = nw for n coincident postsynaptic potentials (PSPs) of size w), this probability distribution is shifted by Δv (Fig. 1E, “signal”). The neuron spikes when the membrane potential exceeds the spike threshold θ, which implements the decision threshold to detect these coincidences over the background. The neuron will respond to coincidences (hits) but also to background activity (false alarms), with some probability called the “hit rate” (HR) and “false alarm rate” (FR). Both rates decrease when the threshold increases. For a given value σ of the noise, HR and FR are linked by a curve named the receiver-operating characteristic (ROC), obtained by varying the threshold. ROC curves are shown in Fig. 1F for a noisy integrate-and-fire neuron with exponentially decaying PSPs, with three noise levels (black curves). The rates are calculated as the probability of firing within one integration time constant τ when the neuron receives a PSP of size Δv (HR) and when it does not (FR). That is, the FR is the product Fτ, where F is the spontaneous firing rate. Each ROC curve is calculated (with numerical simulations) by varying the spike threshold while keeping the same noise level. Higher thresholds correspond to lower rates.

When the noise is very high, this ROC curve is a diagonal (dashed), meaning that coincidences cannot be distinguished from background. As the noise decreases, the ROC curve shifts toward the upper left corner, meaning that spikes indicate coincidences more reliably. In signal detection theory, this relationship between hit rate and false alarm rate is quantified by the *sensitivity index* d′, which, for normal distributions, is the spread between the distributions in units of the noise standard deviation: 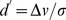. Red curves in Fig. 1F show the theoretical ROC curves for the noise values used in the simulations. Thus, d′ quantifies the ability to detect coincidences while the value of the spike threshold corresponds to a particular trade-off between hit rate and false alarm rate.

For example, in the case of two coincident spikes, one simple choice is θ = 2w (relative to the mean membrane potential), which ensures a HR of 50% when the two input spikes are synchronous, and a lower FR for background activity (Fig. 1F, horizontal dashed line). More generally, the ratio between HR and FR increases when the FR decreases: this implies that, to detect coincidences, the false alarm rate should be set to a low level. For an integrate-and-fire neuron with spontaneous firing rate F and integration time constant τ, we have defined the FR as F.τ. Some experimental evidence indicates that this quantity is indeed low *in vivo*: the membrane time constant is short *in vivo* (e.g. around 5 ms in the frontal cortex [39]), because of the large total synaptic conductance [40]; average firing rates are low, possibly smaller than 1 Hz [41]. Although the latter point is controversial, the product F.τ remains small even with larger estimates of F. In addition, we note that the temporal window of integration is in fact shorter than the membrane time constant, because of spike threshold adaptation [42], [43], and because of coordinated inhibition [44]. This ensures that the ratio HR/FR is high, even for small d′ (small depolarization 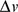).

### Temporal resolution of coincidence detection

Thus, neurons can detect coincidences above background noise, but an important question is the temporal resolution of coincidence detection. We can use signal detection theory again to address this question. Consider two input spikes delayed by a time δ, each one producing an exponential PSP of size w and decay time τ (Fig. 1G). When the spikes are synchronous (Fig. 1G, left), the membrane potential at peak time is 2w, plus the background noise. When they are delayed (Fig. 1G, right), the peak membrane potential is 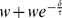, plus the noise. To detect between these two possibilities, we need to distinguish between two random variables with means differing by 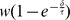 and standard deviation σ (Fig. 1H). This corresponds to a sensitivity index
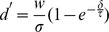
and for short delays (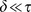): 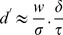. This can be described as the product of the signal-to-noise ratio (w/σ) with the delay in units of the time constant.

We can now define the temporal resolution of coincidence detection using the concept of “just noticeable difference” (JND), defined as the delay 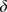 for which spikes can be correctly distinguished from synchronous spikes with 75% probability (assuming 50% correct answers for 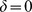). This corresponds to a d′ of 1.35 [35], which gives for short delays:
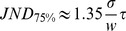
Thus, the temporal resolution of coincidence detection is proportional to the integration time constant 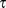, and inversely proportional to the signal-to-noise ratio 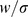. Note that the approximation 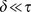 corresponds here to 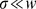, i.e., low noise. The precise expression using the original formula for d′ is:
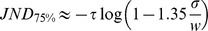
This expression is only defined with relatively low noise, when 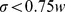: this is because above this value, it is not possible to correctly distinguish between synchronous and asynchronous spikes (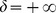) with 75% probability.

Let us come back to the specific example of duration selectivity I have presented above. The postsynaptic neuron receives input spikes from neuron A and neuron B, at latencies L_A_(D) and L_B_(D), where D is the duration of the stimulus. The latency curves intersect at some duration D* (500 ms in Fig. 1B). The timing difference between the two spikes is 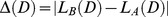. We approximate it near the intersection point as 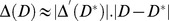, and we obtain this approximate expression of the JND in duration:
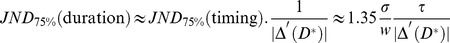
The term 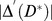 quantifies how different the latency curves are near the intersection point. This formula indicates that the detection of duration is more accurate when the properties of the presynaptic neurons are heterogeneous.

### Decoding synchrony patterns

**Figure 2:**
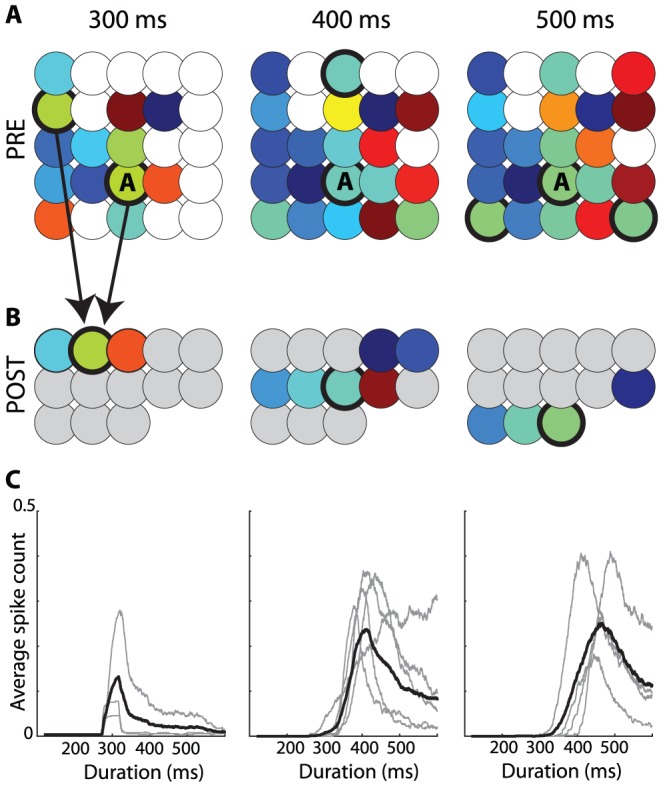
Decoding synchrony patterns in a heterogeneous population. Each column corresponds to one stimulus duration. A, Color represents the latency of the spike produced by each neuron responding to the stimulus (white if the neuron did not spike). Thus, neurons with the same color are synchronous for that specific stimulus (duration). The population can be divided in groups of synchronous neurons (i.e., with the same color), forming the “synchrony partition”. Circled neurons belong to the synchronous group of neuron A. B, Each synchronous group projects to a postsynaptic neuron. Each duration is associated with an assembly of postsynaptic neurons. C, Activation of the postsynaptic assembly as a function of duration (grey: individual neurons; black: average).

We can now apply these principles to decode synchrony patterns at the population level. Consider a population of neurons with rebound spiking properties but heterogeneous parameters. For example, in Fig. 2A, the membrane time constant varies randomly across neurons between 10 and 50 ms, and the K+ channel time constant varies between 300 and 500 ms (see Text S1 for a justification of this choice of parameter values). For a given stimulus, for example an inhibitory pulse with duration 300 ms, we can look at the synchrony pattern in the neural population. In Fig. 1E (top left), neurons represented with the same color produce a rebound spike at the same time (with a 2 ms precision), that is, the stimulus is in the SRF of neurons with the same color. Thus, the neuron population can be divided in groups of synchronous neurons (possibly containing just one neuron). I call this partition of the neural population the *synchrony partition* (mathematically, it is the neural partition defined by synchrony, which is an equivalence relation). This definition mirrors the definition of the SRF: the SRF describes the set of stimuli for which a given group of neurons are synchronous, the synchrony partition describes the groups of neurons that are synchronous for a given stimulus. Fig. 2A shows the synchrony partition in a population of 25 heterogeneous neurons for three stimuli: inhibitory pulses of 300 ms, 400 ms and 500 ms. Each stimulus produces a different synchrony partition: for example, the three neurons colored in green for the 300 ms stimulus are not synchronous for the 400 ms stimulus.

Decoding synchrony patterns is now straightforward (Fig. 2B). For each synchrony partition (each stimulus), we assign a population of postsynaptic neurons, one neuron for each group in the partition (colored neurons in Fig. 2B). Presynaptic neurons in the same group (same color) make excitatory synapses onto the same postsynaptic neuron. In this figure, the peak size of PSPs was set as the difference between threshold and mean potential divided by the number of neurons in the presynaptic group: this choice means that the hit rate should be 50% (only approximately, since input synchrony is not perfect). Therefore, the postsynaptic neural assembly maximally fires for a specific synchrony partition, that is, for a specific stimulus (Fig. 2C). In this way, synchrony partitions are mapped to patterns of postsynaptic activity, and SRFs are mapped to standard receptive fields.

We note in Fig. 2C a few deviations from the ideal scenario described above. First, the maximum firing probability is generally lower than 0.5. This is because a synchronous group was defined as a group of neurons that fire within 2 ms of each other, rather than at the exact same time. With more encoding neurons, groups could be defined with a better precision (i.e., finer synchrony partitions). Second, the duration selectivity curves are non-symmetrical, with more spikes produced at longer durations. This is because there is more heterogeneity in spike latency at short durations (where latency curves diverge, see Fig. 1B) than at long durations (where latency curves are constant). Making the integration time constant of coincidence detectors shorter would reduce this phenomenon. As a consequence of these two facts, selectivity curves do not peak exactly at the expected duration. The ideal scenario corresponds to the limit case where stimuli are encoded by many neurons (allowing fine synchrony partitions) and synchrony patterns are decoded with a fine resolution (short time constant of coincidence detectors).

**Figure 3:**
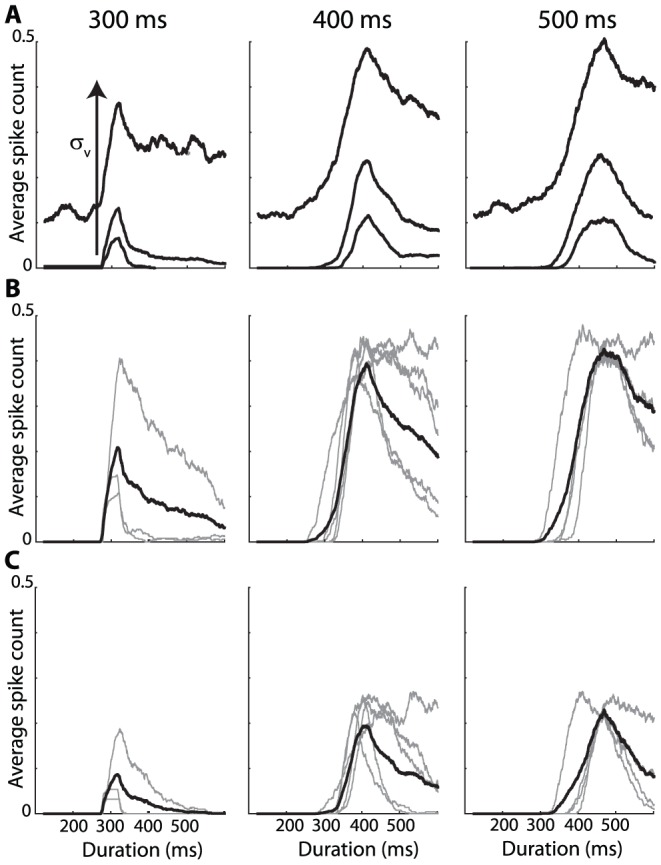
Generality of coincidence detection. A, Activation of postsynaptic assemblies as a function of duration (as in Fig. 2C) for three noise levels: σ_v_ = 0.07, 0.14, 0.28 (bottom to top curve). B, Same as A with synaptic conductances and σ_v_ = 0.14 (as in Fig. 2C; grey: individual neurons; black: average). C, Same as B using neurons with rebound spiking (identical to the presynaptic neurons).

Decoding synchrony patterns requires that neurons are sensitive to coincidences (in the sense that they fire more when their inputs are coincident), but it does not rely on specific neural properties, as is shown in Fig. 3. Varying the amount of internal noise quantitatively changes the neuron sensitivity to coincidences (the sensitivity index d′ in the signal detection theory perspective) but it does not change the qualitative properties (Fig. 3A). In Fig. 3B, inputs to the neurons were modeled as excitatory synaptic conductances (exponentially decaying with time constant τ_e_ = 2 ms). The main difference is that the size of PSPs now depends on the driving force (synaptic reversal potential minus membrane potential). However, as argued in [19], for an excitatory synapse, the driving force is restricted to a rather small range below spike threshold (50–80 mV), so that it has little impact on PSP size and on coincidence detection properties. In Fig. 3C, the coincidence detector neurons are modeled in the same way as the presynaptic neurons, with rebound spiking (with time constants τ = 10 ms and τ_KLT_ = 400 ms, see the Methods for details). That is, neurons of the same type encode the stimuli and decode the synchrony patterns. The results are qualitatively unchanged.

### Learning synchrony codes

I have shown an explicit construction of the decoding circuit, but how can this circuit spontaneously emerge?

As explained above, a simple condition for a neuron to be sensitive to coincidences is to ensure that its firing rate is low. This can be implemented by a homeostatic principle. Two physiologically plausible mechanisms are intrinsic plasticity, where excitability (e.g. spike threshold or membrane resistance) changes with activity [45], and synaptic scaling, where synaptic weights change with pre- and/or post-synaptic activity [46]. In the context of signal detection theory (Fig. 1E–H), homeostasis can be seen as the process of setting the decision threshold so as to maintain a low false alarm rate. I consider a simple synaptic scaling mechanism in which synaptic weights continuously increase, independently of pre- and post-synaptic activity, and each postsynaptic spike reduces all synaptic weights:
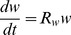


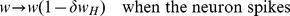
This multiplicative form corresponds to experimental observations [47] and it also has theoretical advantages: 1) it is equivalent to a change in spike threshold, 2) it leaves the relative strengths of the synapses unchanged and 3) it keeps the weights positive, without imposing a hard boundary. Weights are stable when 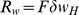 (where F is the postsynaptic firing rate), that is, when 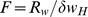. Thus this mechanism maintains a target firing rate F.

**Figure 4:**
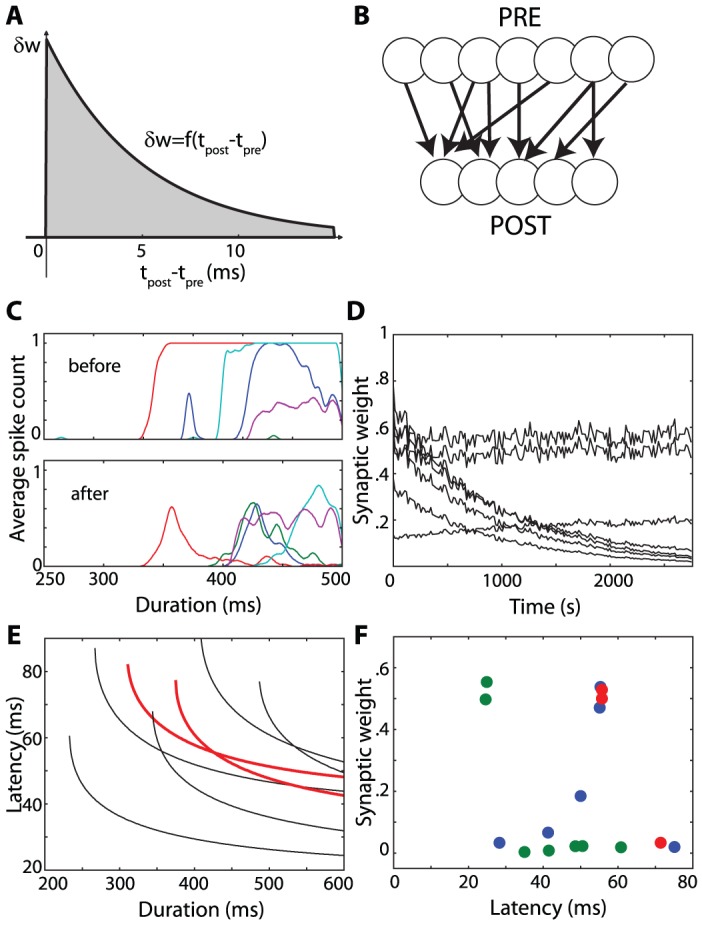
Learning in the duration model. A, In addition to homeostasis, synaptic weights are modified by 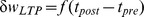 for every pair of pre and postsynaptic spikes at times t_pre_ and t_post_, respectively. B, Presynaptic neurons project to random postsynaptic neurons, with on average 5 synapses per postsynaptic neuron. C, Duration selectivity curves for 5 postsynaptic neurons at the beginning (top) and end (bottom) of the learning period. D, Temporal evolution of the synaptic weights of the neuron corresponding to the blue curves in C. E, Spike latency as a function of stimulus duration for all the presynaptic neurons of the postsynaptic neuron selected in D. Red curves correspond to the two strongest synapses. F, For three postsynaptic neurons (colors as in C), synaptic weights are shown against spike latency of the corresponding presynaptic neurons, at the best duration of the postsynaptic neuron.

Homeostasis acts on the decision threshold but is not synapse-specific (that is, it does not improve the sensitivity index d′). In the circuit shown in Fig. 2, the postsynaptic neuron fires when the presynaptic neurons belong to the same (stimulus-specific) synchronous group. To develop such circuits requires a synaptic plasticity mechanism that selectively strengthens synapses that are co-activated with the postsynaptic neuron, in a short temporal window corresponding to the precision of the synchrony partition. This is consistent with the properties of long-term potentiation in spike-timing-dependent plasticity (STDP) seen at excitatory synapses onto excitatory neurons [48], and theoretical studies have shown that STDP favors correlated inputs [20], [21], [49]. In addition to homeostasis, I consider an STDP rule in which the synaptic weight modification 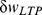 depends on the difference in timing t_post_-t_pre_ of a pre- and post-synaptic spike (Fig. 4A):
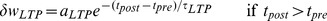



The synaptic modifications induced by all pairs of pre and post spikes are added, but in this context where firing rates are low (around 1 Hz), the precise way in which pairs interact does not make a difference. The time constant 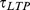 is set equal to the membrane time constant τ. I also choose a small value for a_LTP_, so that the average firing rate is mainly determined by the homeostatic mechanism while the relative strengths of synapses are determined by the correlations between the synaptic inputs and the neuron output. It is not necessary to impose a boundary on the synaptic weights, because stability is ensured by the homeostatic mechanism. In the same way, long term depression (LTD) is unnecessary here, and it is ignored for simplicity.

I consider a group of presynaptic neurons (100 were simulated) and postsynaptic neurons as in Fig. 2, connected by random synapses, with an average of 5 synapses per postsynaptic neuron (Fig. 4B). The synaptic weights are initially random between 0 and 1 (1 is the spike threshold), and they evolve through homeostasis and STDP while 5000 stimuli with random duration are sequentially presented. Fig. 4C shows the selectivity curves of 5 postsynaptic neurons, before (top) and after learning (bottom), as in Fig. 2C. Initially, neurons tend have high-pass properties, that is, they fire when the stimulus is longer than a given duration. This mirrors the properties of the inputs (Fig. 1B). In one case (green curve), the neuron almost never fired to any stimulus. After learning, most neurons have a peaked selectivity curve, with a preferred duration. Fig. 4D shows the evolution of synaptic weights during learning for the postsynaptic neuron corresponding to the blue curves in Fig. 4C. It appears that most synaptic weights decay, except two of them which stabilize at 0.5 (half the distance to spike threshold) and one weaker synapse. The properties of these synapses are shown in Fig. 4E. Each curve represents the spike latency of the presynaptic neurons for the neuron considered in Fig. 4D (as in Fig. 1B), and are the two strongest synapses are displayed in red. It appears that these two curves intersect at a duration of about 430 ms, which is the best duration of the neuron shown in blue in Fig. 4C. This illustrates the idea that the postsynaptic neuron fires when the stimulus is in the synchrony receptive field of its presynaptic neurons. Fig. 4F shows that the learning mechanism selects synapses in the same way as I described in Fig. 2, that is, it selects synapses that are synchronously active for a specific stimulus duration. Each color corresponds to a postsynaptic neuron (same color code as in Fig. 4C) and each dot represents the weight of one synapse vs. the spike latency of the corresponding presynaptic neuron, at the best duration of the postsynaptic neuron. For example, for the green neuron, the two strongest synapses are synchronously active (same spike latency) at the best duration (about 420 ms, Fig. 4C), while the other synapses are activated at diverse latencies. Similar observations can be made for the two other neurons. An interesting point is that the blue and green neurons have the same best durations (about 420–430 ms, Fig. 4C) but respond at different latencies (about 25 ms and 55 ms; strongest synapses in Fig. 4F). This corresponds to two different groups of the synchrony partition in Fig. 2 (neurons shown with two different colors in the same column).

Thus, the proposed decoding circuit (Fig. 2) can emerge in an unsupervised way, through a combination of homeostasis and STDP.

### Stimulus-dependent synchrony in sensory modalities

**Figure 5:**
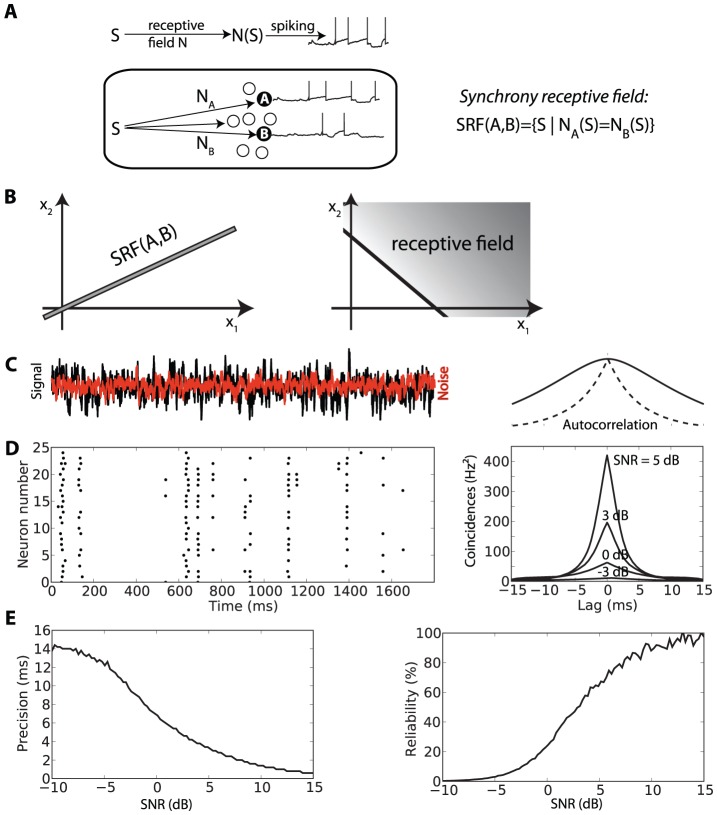
Synchrony mechanism with sensory stimuli. A, Schematic representation of stimulus encoding by a neuron: the stimulus S is filtered through the receptive field N, and the resulting signal N(S) is nonlinearly transformed into spike trains. The synchrony receptive field of two different neurons A and B is the set of stimuli such that the two filtered signals match: N_A_(S) = N_B_(S). B, Schematic representation of a standard receptive field (N(S)>θ) and a synchrony receptive field in a two-dimensional world. C, Fluctuating input and independent noise. Right: input autocorrelation (time constant 5 ms). D, Responses of a noisy integrate-and-fire model in repeated trials. Right: shuffled auto-correlogram (SAC) for different signal-to-noise ratios (SNR). E, Precision and reliability of spike timing as a function of SNR.

I introduced the concepts of synchrony receptive fields and synchrony partition with an elementary example, duration selectivity, where stimuli are one-dimensional. Real world stimuli, on the other hand, vary along many dimensions, which makes computation much more difficult [50]. To understand synchrony patterns in this more general setting, I describe neuron responses in the following simplified way (Fig. 5A, top): a stimulus S is transformed through a linear or non-linear filter N, which represents the (standard) receptive field of the neuron, then the filtered stimulus N(S) is mapped to a spike train through a non-linear spiking transformation (for example, N(S) is the input to a spiking neuron model). Note that although this description appears to be feedforward, the computation of the filter N may or may not rely on a feedforward circuit. Assuming that two neurons A and B fire in synchrony when they receive the same dynamic input N_A_(S) and N_B_(S), the SRF of A and B is the set of stimuli S such that N_A_(S) = N_B_(S). In mathematical terms, this is a manifold of stimulus space; if the neural filters are linear, it is a linear subspace of stimuli. For example, in two dimensions, the SRF is a line (Fig. 5B, left). In contrast, a neuron fires when the filtered stimulus exceeds some threshold, N(S)>θ, that is, in two dimensions, when the stimulus is on one side of a line (Fig. 5B, right). In higher dimension, a neuron fires when the stimulus is on one side of a hyperplane, while two neurons fire in synchrony when the stimulus is close to a hyperplane (assuming linear filtering). This makes computation with synchrony qualitatively different from rate-based computation, with interesting computational properties, for example SRFs are unchanged by linear scaling of the stimulus (i.e., intensity change).

I will describe these qualitative differences in more details in the next section, but first I will comment on the hypothesis that two neurons fire in synchrony when they receive the same dynamical input. First, this should not be true if the neurons have different intrinsic properties (for example, spike threshold or resistance). Therefore I consider that the heterogeneity in intrinsic properties is implicitly included in the description of the receptive field (or filter) N. For example, the membrane resistance can be included as a gain applied to the filter N (N→R.N) rather than in the spiking transformation; the membrane time constant can be included as a low-pass filter. Thus the hypothesis really means that two identical neurons fire in synchrony in response to identical time-varying stimuli. *In vitro* experiments have demonstrated that a single cortical neuron responds identically (at a millisecond timescale) to repeated time-varying currents [51]. As for coincidence detection properties, the main condition is that the neuron is in a fluctuation-driven regime, with a subthreshold average input [52], [53]. This property is illustrated with neuron models in Fig. 5C–E, which shows the response of a spiking neuron model to a fluctuating input (Fig. 5C) over repeated trials, with a subthreshold mean. The same current is presented in all trials, with an additional independent noise (red). This noise represents both the intrinsic noise and the difference in inputs between trials. If the noise level is low enough, spike timing is reproducible at a fine timescale, as shown by the shuffled autocorrelogram (SAC, see [54]) (Fig. 5D, right). A very important property is that the precision of synchrony between trials, as estimated by the width of the SAC (Fig. 5E; see Methods), reflects the similarity of the input signals (measured by the signal to noise ratio), rather than the intrinsic timescale of the signal fluctuations (seen in the autocorrelation of the signal in Fig. 5C, right). In particular, when noise level goes to 0, precision converges to 0 ms rather than to the timescale of input fluctuations (Fig. 5E, left). Therefore, when two identical neurons receive inputs N_A_(S) and N_B_(S), their degree of synchrony reflects the degree of similarity between N_A_(S) and N_B_(S). This is related to the mechanism used by Brody and Hopfield [55], [56] in a previous model of odor recognition based on spike timing, where constant inputs are added to an external oscillation, but it is more general. That oscillation-based mechanism works only in a limited input range (see Fig. 1 in [55]) because it relies on 1∶1 phase-locking (one spike per period of the oscillation) in a mean-driven regime (average input above threshold), which is less robust than the mechanism shown here [53] (phase locking is also more robust in the fluctuation-driven regime [57]).

**Figure 6:**
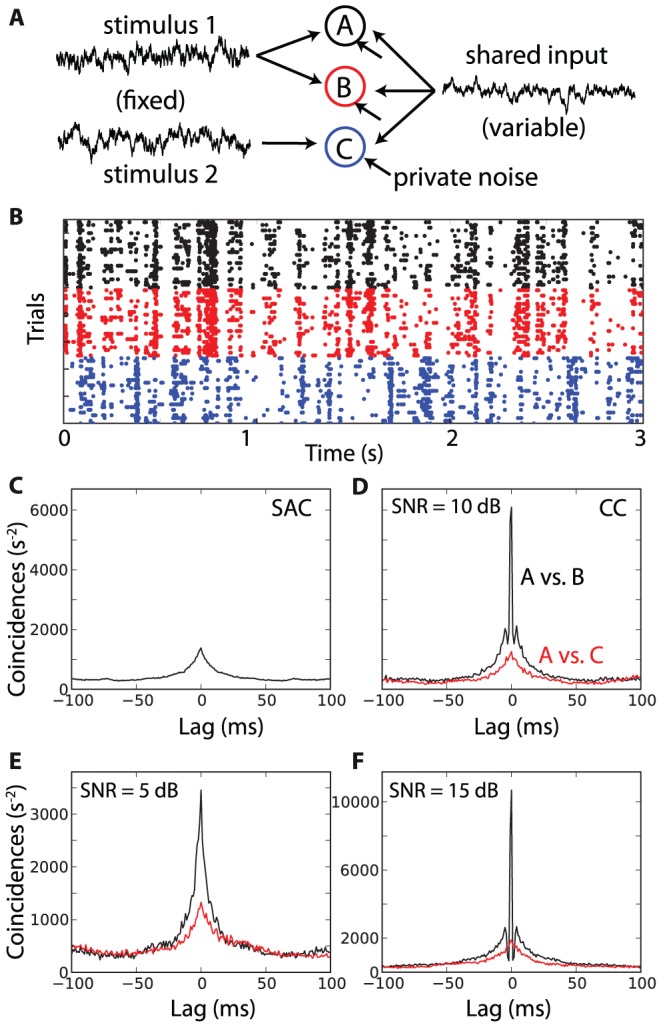
Synchrony without trial-to-trial reproducibility. A, Neurons A and B receive the same stimulus-driven input, neuron C receives a different one. The stimuli are identical in all trials but all neurons receive a shared input that varies between trials. Each neuron also has a private source of noise. B, Responses of neurons A (black), B (red) and C (blue) in 25 trials, with a signal-to-noise ratio (SNR) of 10 dB (shared vs. private). C, The shuffled autocorrelogram of neuron A indicates that spike trains are not reproducible at a fine timescale. D, Nevertheless, the average cross-correlogram between A and B shows synchrony at a millisecond timescale, which does not appear between A and C. E, Same as D with SNR = 5 dB (note the different vertical scale). F, Same as D with SNR = 15 dB.

This reproducibility of spike timing has been demonstrated *in vitro* [51] and *in vivo* in early sensory pathways such as the retina [5] and the auditory brainstem [58], but it could be argued that it is an unrealistic assumption in other neural structures. However, synchrony-based computation does not critically rely on reproducible spike timing but rather on reproducible synchrony. Specifically, network activity may introduce inter-trial variability that is shared by neurons, as seen in the auditory cortex [59], degrading the reproducibility of absolute spike timing but not of relative spike timing. This is shown in Fig. 6, where three model neurons receive a stimulus-driven input, identical in all trials, and a shared external input, variable between trials. In addition, each neuron has a private source of noise. Neurons A and B receive the same stimulus-driven input, meaning the stimulus is in the SRF of A and B, and neuron C receives a different input (Fig. 6A). It appears that spike-timing reproducibility is low for all neurons (Fig. 6B,C), but that A and B are reliably synchronized in all trials (Fig. 6D, cross-correlogram). The peak of the cross-correlogram depends on the signal-to-noise ratio, defined between the shared and private components of the noise (Fig. 6E,F). This dependence can be quantified in exactly the same way as in Fig. 5E, where the signal is the sum of the stimulus and of the shared noise, while the noise corresponds to the private noise. Therefore, the mechanism used here does not critically rely on reproducible spike timing, but rather on reproducible stimulus-dependent synchrony.

### Structure and synchrony

**Figure 7:**
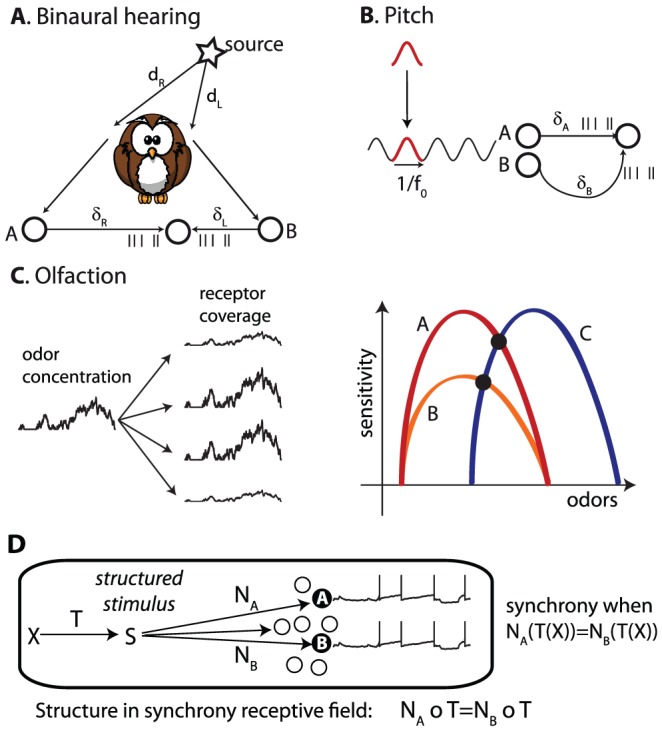
Structure and synchrony. A, Binaural hearing (simplified). The sound arrives at the two ears after a propagation delay d_L_ and d_R_. Monaural neurons A and B project to a binaural neuron with axonal conduction delays δ_L_ and δ_R_. Synchrony (seen on the postsynaptic side) occurs when d_R_−d_L_ = δ_L_−δ_R_, corresponding to a specific interaural time difference. B, Pitch. Two monaural neurons responding to a sound project to a postsynaptic neuron with axonal delays δ_A_ and δ_B_. From the postsynaptic point of view, synchrony occurs for a periodic sound with period 1/f_0_ matching the delay difference: 1/f_0_ = δ_B_−δ_A_. C, Olfaction. Left, Odor concentration fluctuates rapidly because of turbulences, and odorant molecules bind to different types of receptors. Each receptor has an odor-specific affinity, so that its coverage by the odor is the product of concentration and affinity. Right, Olfactory neurons A and B have the same receptor type but different global sensitivities, neuron C has a different receptor type. Colored curves schematically represent the sensitivity to different odors, defined as the product of odor affinity and global sensitivity. Synchrony occurs at intersection points, for specific odors. D, More generally, a structured stimulus is described as the image of a lower-dimensional stimulus X through some transformation T. Synchrony occurs in two different neurons when their receptive fields match when combined with the transformation T.

In this framework, a random stimulus cannot produce tightly synchronous responses in neurons with different receptive fields. Therefore, synchrony must reflect some non-randomness or “structure” in the stimulus. Fig. 7 illustrates the relationship between synchrony and structure with a few sensory examples.

A classical example is binaural hearing (Fig. 7A). Leaving sound diffraction aside for the moment (see last section of the Results), the sound S(t) produced by a source on the left of the animal will arrive first at the left ear, then at the right ear, with propagation delays d_L_ and d_R_, respectively. Therefore the two monaural signals are S_L_(t) = S(t−d_L_) and S_R_(t) = S(t−d_R_), respectively. The interaural time difference ITD = d_R_−d_L_ depends on the azimuth of the source. The binaural stimulus (S_L_, S_R_) has a structure, in that both S_L_ and S_R_ are transformations of the same signal. That structure is specific of a particular ITD.

Consider two monaural neurons A and B on opposite sides, which project to a binaural neuron with axonal delays δ_L_ and δ_R_. From the postsynaptic point of view, the SRF of A and B should include the axonal conduction delays. It is the set of stimuli (S_L_, S_R_) such that S_L_(t−δ_L_) = S_R_(t−δ_R_), that is: S_L_(t) = S_R_(t−(δ_R_−δ_L_)). Therefore, the SRF of A and B is the set of all binaural signals produced by a single source with ITD δ_L_−δ_R_, and it is independent of the source signal. Thus, the SRF indicates the structure of the stimulus, an information that is not present in the individual responses of the monaural neurons. The binaural neuron depicted in Fig. 7A fires when the stimulus is the in SRF of A and B, that is, at a specific source location. This is in essence the Jeffress model of sound localization [60].

Similar concepts apply to pitch perception (Fig. 7B). Pitch is the perceptual correlate of the periodicity of sounds, such as vowels or musical notes (to a first approximation). A periodic sound S(t) can be described as the repetition of a signal defined on one cycle (red curve). The repetition rate f_0_ determines the pitch, while the original signal determines the timbre. As for the binaural example, this produces a specific structure in the signal S(t), and the structural information is associated with the pitch of the sound. Consider two neurons A and B with the same properties but different axonal delays δ_A_ and δ_B_. The SRF of A and B (again from the postsynaptic side, including axonal delays) is the set of signals such that S(t−δ_A_) = S(t−δ_B_). These are all the periodic signals with repetition rate f_0_ = 1/|δ_B_−δ_A_|, or a multiple of it. Again synchrony reflects a structural property of the stimulus. In essence, this is Licklider's model of pitch perception [61].

The third example is olfaction (Fig. 7C). There is considerable heterogeneity in the properties of olfactory sensory neurons: there are about 1000 receptor types in rats, and neurons which express the same olfactory receptor type respond to the same odorants but vary in global sensitivity, up to 100-fold [62]. Odor plumes are highly turbulent [63], so that their concentration c(t) in the olfactory epithelium varies very quickly (Fig. 7C, left). The receptor coverage is defined as the probability that the receptor is bound to the odorant molecules. It can be expressed as *a^O^.c(t)*, where *a^O^* the affinity of the receptor type to the presented odor *O*. Thus the olfactory stimulus can be represented as 1000 time-varying signals (receptor coverage for all types), but these signals have a strong structure since they are all scaled versions of the same signal (odor concentration c(t)). Olfactory neurons of the same type differ in their global sensitivity *s*, so the activation of an olfactory neuron is essentially determined by the product of concentration *c(t)*, odor affinity *a^O^* (type-specific and odor-specific) and global sensitivity *s* (neuron-specific): *c(t).a^O^.s* (the transformation of this signal to spike trains is highly nonlinear). Fig. 7C (right) schematically represents the value *a^O^.s* as a function of odor identity for three neurons: neurons A and B respond to the same odors (same receptor type), but A has higher global sensitivity than B; neuron C responds to different odors (different type). Tuning is broad, so a given odor elicits responses in many different olfactory neurons. The SRF of A and C is the set of olfactory stimuli such that c(t).a_A_ *^O^*.s_A_ = c(t).a_C_ *^O^*.s_C_: the product of odor affinity and sensitivity is the same for neurons A and C. Although odor concentration c(t) varies very quickly, the identity a_A_ *^O^*.s_A_ = a_C_ *^O^*.s_C_, which defines the SRF of A and C, does not depend on it. In Fig. 7C, the SRF of A and C is the single odor at the intersection of the two tuning curves. Neurons B and C have a different SRF since their tuning curves intersect at a different odor. I will make this example more specific in the next sections.

In all these examples, synchrony patterns reflect the structure of the stimulus. This idea can be formalized by describing a structured stimulus S as the image of a lower-dimensional object X through some transformation T: S = T(X) (Fig. 7D). In the binaural hearing example, X is the source signal, S = (S_L_,S_R_) is the binaural signal, and T is the acoustical transformation: T(X) = (X(t−d_L_),X(t−d_R_)). In the olfactory example, X is the time-varying concentration c(t), S is the time-varying coverage of all receptors (a time-varying 1000-dimensional vector), and T is the transformation c(t)→**a^O^**.c(t), where **a^O^** is the vector of affinities of all receptor types to the presented odor. Table 1 describes other examples in this framework. This structure introduces synchrony in all neurons whose receptive fields match when combined with the transformation T: N_B_ ∘ T = N_A_ ∘ T (composition of mappings), where N_A_ and N_B_ are the receptive fields of the two neurons. In the olfactory example, this means that the product of odor affinity and sensitivity is the same for neurons A and B (a_i_^O^.s_i_ = a_j_^O^.s_j_); in the binaural hearing example, this means that the combination of acoustical and neural delays match on both sides.

**Table 1:** Structure and synchrony in sensory modalities.

	Binocular disparity	Binaural hearing I	Binaural hearing II	Pitch	Visual edges	Olfaction
**X (carrier)**	Visual object	Source signal S(t)	Source signal S(t)	One period of a sound	Intensity pattern along one dimension	Concentration c(t)
**T (structure)**	Projection to two retinal images	Acoustical propagation delays	Acoustical filtering (HRTFs)	Repetition	Repetition along another dimension	Binding of odor to all receptor types: T(X) = **a**.c(t)
**Receptive fields N**	Circular receptive fields	Axonal conduction delays	Auditory filters (include delay)	Auditory filters (include delay)	Circular receptive fields	Receptor type and global sensitivity: N_i_(T(X)) = a_i_.s_i_.c(t)
**SRF**	3D visual point	Specific ITD	Specific filter pair (source location)	Periodic sounds with specific period, or resolved harmonics	Edges with specific orientation	Specific odor

This identity defines a synchrony partition that reflects the structure of stimuli (induced by the transformation T), independently of the source X (e.g. the time-varying concentration). This is an appealing property from a computational point of view, because stimulus structure has natural invariances: for example, binaural structure depends on source location, but not on source signal; in olfaction, structure is independent of concentration. These invariances appear in the synchrony partitions, even though neurons have heterogeneous properties and their individual responses vary with many aspects of stimuli. We now look at the computational properties of these structural codes, taking the example of olfaction.

### Computing with synchrony: olfaction

**Figure 8:**
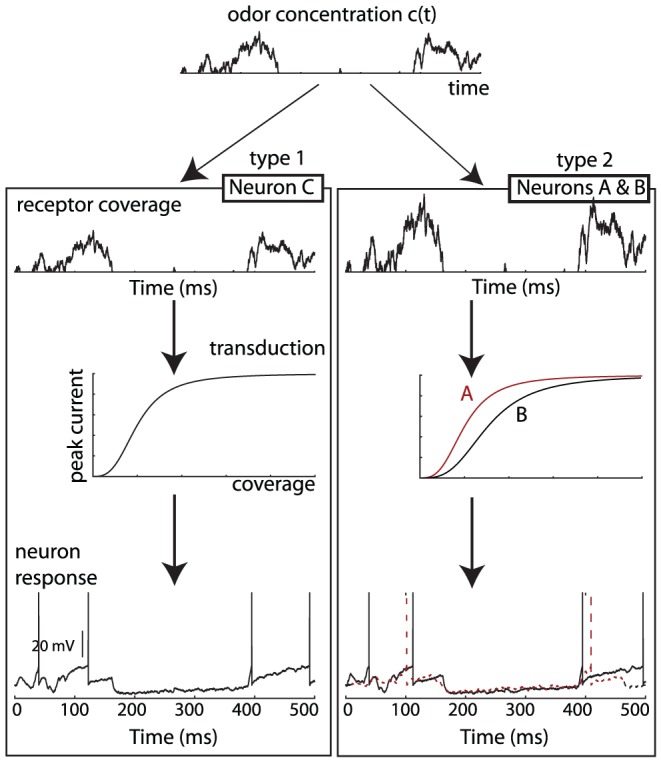
Synchrony receptive fields in an olfactory model. Top, An odor is presented with fluctuating concentration c(t). Receptor coverage is the affinity of the receptor type (type 1 for neuron C, type 2 for neurons A and B) times the concentration: a.c(t). The peak transduction current (middle) is a Hill function of receptor coverage, with different half-activation coverage for different neurons (inverse of global sensitivity). Neurons fire in synchrony to an odor when the product of odor affinity and global sensitivity match. This occurs for neurons B and C (black traces), but not for neurons A and C (dashed red trace).

Fig. 8 shows odor-specific synchrony in a simple olfactory model, corresponding to the situation shown in Fig. 7C, with an odor in the SRF of neurons B and C. Odor concentration c(t) varies randomly with turbulences, and receptor coverage depends on concentration and receptor type: receptor type 2 (neurons A and B) is more sensitive to the presented odor than receptor type 1 (neuron C). The odor is then transduced into a current, which produces spikes. The transduction current is modeled as a Hill function of receptor coverage: I = I_max_*H_n_(s.c) (Fig. 8, middle), where *I_max_* is the maximum current, *c* is the odor coverage, s is the global sensitivity (inverse of the half-activation coverage) and n is the Hill coefficient, related to the slope of the curve [64]. The Hill coefficient is not very variable, but *s* can vary 100-fold among olfactory sensory neurons expressing the same olfactory receptor: here, neuron B has a higher sensitivity than neuron A. Thus, the transduction current is essentially determined by the quantity a.s, where a is the affinity of the receptor type to the presented odor. Here, neuron B has a higher affinity to the odor than neuron C, but its global sensitivity is lower, so that the transduction current is the same. As a result, the neurons fire in synchrony (black traces in Fig. 8, bottom; neurons were modeled as integrate-and-fire models). On the other hand, neuron A has the same global sensitivity as C but different affinity and thus does not fire in synchrony (red dashed trace). Synchrony is independent of odor concentration.

**Figure 9:**
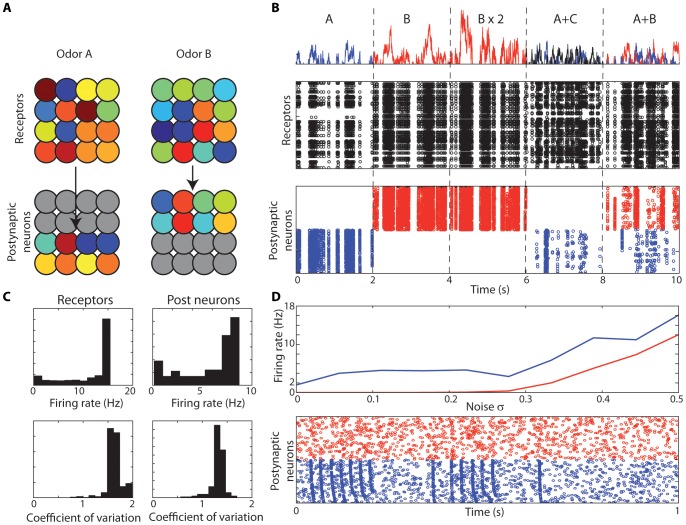
Computing with synchrony in olfaction. A, Top, Different odors produce different synchrony partitions (receptors with the same color are synchronous). Bottom, To each odor corresponds an assembly of postsynaptic neurons, where the inputs to each neuron belong to the same synchrony group (in each column, each postsynaptic with a given color receives synapses from all receptors with the same color). B, Top, Fluctuating concentration of three odors (A: blue, B: red, C: black). Middle, spiking responses of olfactory receptors. Bottom, Responses of postsynaptic neurons from the assembly selective to A (blue) and to B (red). Stimuli are presented is sequence: 1) odor A alone, 2) odor B alone, 3) odor B alone with twice stronger intensity, 4) odor A with distracting odor C (same intensity), 5) odors A and B (same intensity). C, Spike train statistics for the receptors (left column) and the postsynaptic neurons selective for odor A (right column), corresponding to the stimulation in the first 2 seconds of panel B. Top, distribution of firing rates; bottom, distribution of coefficients of variation. D, Top, Average firing rate in the assembly of postsynaptic neurons selective to A (blue) and in the assembly selective to B (red) when odor A is presented (as in panel B, first two seconds), as a function of the intrinsic noise (standard deviation relative to spike threshold). Bottom, Responses of the postsynaptic neurons for the maximum amount of intrinsic noise (σ = 0.5).

Let us now consider a population of olfactory neurons (Fig. 9). Each odor is represented by a random vector of affinities and odor concentration is modeled as a half-wave rectified low-pass filtered noise. Receptors and postsynaptic neurons are noisy integrate-and-fire models with random global sensitivity (see Methods). Each odor induces a specific synchrony partition in receptors (Fig. 9A, top). The color represents the product of their odor affinity and global sensitivity, therefore as in Fig. 2, two receptor neurons with the same color fire in synchrony to the presented odor. These patterns can be decoded by postsynaptic neurons, which receive inputs from neurons in the same synchrony group (Fig. 9A, bottom). When odor A is presented (Fig. 9B, first column), postsynaptic neurons wired to the specific synchrony pattern of odor A fire. When odor B is presented, the corresponding postsynaptic neurons fire (Fig. 9B, second column), but neurons tuned to A do not fire, because they do not see synchronous inputs. On the other hand, most receptor neurons fire in both cases, because of their broad tuning.

One interesting aspect of mammalian olfaction is that mammals can recognize odors at concentrations that they were not previously exposed to [65]. This invariance to odor intensity is also a natural property of synchrony-based computation, because synchrony receptive fields are invariant to intensity (Fig. 9B, third column), that is, the synchrony partitions (Fig. 9A, top) do not change when intensity varies, even though individual neural responses may change. This simply reflects the fact that the structure of the stimulus (constant ratios of time-varying coverage of different receptor types, as shown in Fig. 7F and 8), which is encoded by synchrony partitions, is intrinsically concentration-invariant.

Another interesting computational property is noise tolerance. When a distracting odor is presented at the same intensity as the target odor, postsynaptic responses are reduced but still odor-specific (Fig. 9B, fourth column). The firing rate is reduced because noise reduces the probability of coincidences, but noise does not increase firing in other odor-specific assemblies, because these neurons receive incoherent inputs. Indeed, by construction, postsynaptic neurons fire when they see coincidences that are unlikely to be caused by chance. Therefore, false alarms (firing of neurons tuned to B) are rare, while neurons tuned to A fire when the signal-to-noise ratio is high enough. This is similar to a strategy described as “listening in the dips” in speech recognition in noise [66]. When two known odors are simultaneously presented, both can be recognized by this principle (Fig. 9B, last column). It should be stressed the reduction in firing rate of the neurons tuned to A when A+C or A+B is presented is not due to an inhibitory mechanism. There is no inhibition in this model. Instead, it is due to a desynchronization of the inputs caused by the distracting odor. A neuron tuned to an odor responds less when another odor is added because the operation that the neuron performs is detecting similarity between sensory signals rather than adding them. For example, suppose the stimulus produces two sensory signals x_1_ and x_2_, and the postsynaptic neuron fires when x_1_ = x_2_. If another stimulus (y_1_, y_2_) is added and the neuron is not tuned to it (y_1_≠y_2_), then x_1_+y_1_≠x_2_+y_2_ and the neuron does not respond. This reduction in firing rate occurs even though all presynaptic receptors fire more (i.e., x_1_+y_1_>x_1_ and x_2_+y_2_>x_2_).

Fig. 9C shows the distribution of firing rates and coefficients of variation in the receptors and postsynaptic neurons, when odor A is presented. The peak in the firing rate distribution indicates that many receptors saturate. As previously discussed (Fig. 1), the sensitivity of postsynaptic neurons to coincidences depends on the level of intrinsic noise. In Fig. 9B, the noise level was σ = 0.15 (relative to the spike threshold). In Fig. 9D, the noise level was varied between 0 and 0.5 and the model was presented with odor A (as in Fig. 9B, first column). The top graphs show the average firing rate of the postsynaptic neurons tuned to A (blue) and to B (red) as a function of noise level. It appears that only neurons tuned to A respond when the noise level is lower than about σ≈0.3. The bottom panel shows the responses of both groups for the highest noise level (σ = 0.5). We note that, although the firing rates of both groups are similar, the temporal structures are very different - that is, the responses of neurons tuned to A are more coherent.

**Figure 10:**
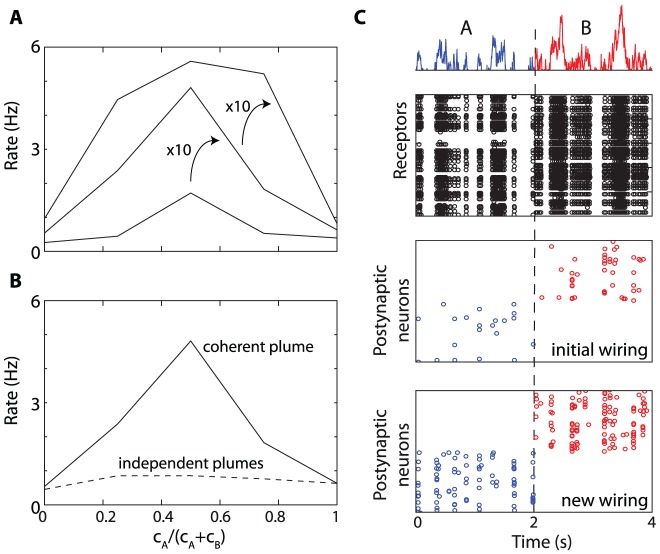
Recognition of odor mixtures and robustness. A, Average firing rate of the postsynaptic assembly tuned to an equal mixture of odors A and B, as a function of the proportion of A in the presented mixture. Each curve corresponds to a different concentration (1, 10, 100). B, Binding: tuning curve of the postsynaptic assembly (same as in A for concentration 10) for mixtures presented in a single turbulent plume (solid) or in two independent plumes for the two odors (dashed). C, Same as in Fig. 6, but the membrane time constant of receptors is heterogeneous (between 15 and 25 ms). With the same synaptic projections as in Fig. 6 (initial wiring), the postsynaptic rate is reduced, but not odor specificity. The firing rate increases when the synaptic projections are adapted to this heterogeneity, i.e., presynaptic neurons have similar membrane time constants (new wiring).

In Fig. 10A, I consider a mixture of two odors A and B and a postsynaptic assembly tuned to the equal mixture (50% A, 50% B). The average firing rate varies with the concentrations of both odors in the mixture and in contrast with Fig. 9B, the presented mixture is always highly correlated with the target mixture. Fig. 10A shows that the assembly responds best when there is an equal proportion of A and B in the mixture, at all concentrations (varying by a factor 100). Although selectivity is broader at the highest concentration, the assembly still responds more to the target mixture at the lowest concentration than to either odor A or B at the highest concentration (×100). Odors A and B are bound into a single mixture because their fluctuations are coherent. If the same odors are simultaneously presented but as a mixture of two independent plumes with their own fluctuations (two different turbulent flows representing two different odor sources), then the network does not bind them together and the assembly does not respond (Fig. 10B). Thus the model implements the idea of binding by synchrony, where precise spike timing acts as a “signature” of an object [24]. More precisely, since neural responses follow the temporal structure of the stimulus, precise coincidences can only detected between neurons that respond to the same stimulus. This is a weak version of binding by synchrony, in the sense that the temporal “signature” is intrinsic to the stimulus rather than created as a result of object formation.

One interesting aspect of synchrony-based computation is that it assigns a functional role to the variability of individual neural properties – here, the variability in sensitivity of olfactory neurons. I have not considered variability in other neural parameters, which may be an issue. In Fig. 9, all receptors had the same membrane time constant (20 ms). If it is made heterogeneous (Fig. 10C, τ = 15–25 ms) and the synaptic projections are unchanged, then postsynaptic neurons see fewer coincidences and fire less (Fig. 10C, initial wiring). This affects the rate but not the specificity of the responses. We may redefine the synaptic projections to take this heterogeneity into account: for example, in Fig. 10C (new wiring), for each postsynaptic neuron, we only choose presynaptic neurons with membrane time constants that differ by less than 5 ms (as well as similar sensitivity to the target odor). As a result, the firing rate is increased and the specificity is unchanged.

**Figure 11:**
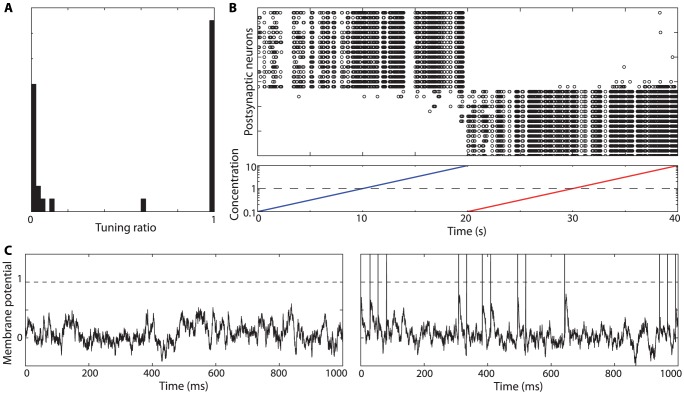
Learning to detect odors. A, Two odors are randomly presented to the network for 40 s. This histogram represents the distribution of tuning ratios after this learning period. The tuning ratio of a postsynaptic neuron is the proportion of spikes triggered by the first odor. B, Responses of postsynaptic neurons, ordered by tuning ratio, to odor A (blue) and odor B (red), with an increasing concentration (0.1 to 10, where 1 is odor concentration in the learning phase). C, Voltage traces for a postsynaptic neuron tuned to odor B, when odor A (left) and B (right) are presented.

Finally, the specific wiring I have described can be learned by synaptic plasticity mechanisms, as explained for the duration model (Fig. 4). In Fig. 11, the two odors A and B were randomly presented to the olfactory model, with random synapses between receptors and postsynaptic neurons (50 synapses per postsynaptic neuron). The presented odor is updated every 200 ms, for a total duration of 40 s. The synaptic weights evolve according to the same homeostatic and synaptic plasticity mechanisms as for the duration model (Fig. 4). At the end of the stimulation, a tuning ratio is calculated for each neuron, as the proportion of spikes in response to odor A, over the second half of the stimulation. That is, a tuning ratio of 0 means that the neuron only responds to odor A, while a tuning ratio of 1 means that it responds only to odor B. Fig. 11A shows the distribution of tuning ratios of the postsynaptic neurons. All neurons but one have tuning ratios clustered near 0 or 1, that is, they are tuned to a single odor. The neurons are then ordered by tuning ratio, and they are presented with odor A with an increasing concentration, then with odor B (Fig. 11B). The concentration varies between 0.1 and 10 (bottom), where 1 is the concentration during the learning phase. It appears that odor selectivity is preserved at all tested concentrations. Fig. 11C shows the voltage traces of a neuron tuned to odor B, when odor A (left) and B (right) are presented (spikes are added for readability). The membrane potential has standard deviation 0.17 (odor A) and 0.18 (odor B, calculated without the spikes), and mean 0.08 (A) and 0.07 (B). Thus, the membrane potential distributions are similar for the preferred and non-preferred odors: the increased firing is due to transient synchrony events rather than changes in input statistics.

This olfactory model shares a few ideas with a spike-timing-based model previously proposed by Brody and Hopfield [55], in particular, odor-specific neurons detect an equality between different quantities by means of synchrony detection. There are a few differences: 1) in the Brody-Hopfield model, the input to encoders is a constant signal, 2) this constant is a logarithmic function of concentration, 3) it is translated into phase by an intrinsic oscillation. The model I have presented has conceptual similarities, but makes weaker hypotheses. First, the input is time-varying instead of constant. Second, the transformation between concentration and input current must be similar across receptors, but it can have an arbitrary form. Third, the transformation from signal to spike times does not rely on an intrinsic oscillation but on the input signal itself. It is less restrictive, because 1∶1 phase-locking occurs under specific conditions. However, adding an internal oscillation to the stimulus-locked signal would also work in the present model, if it is shared across encoding neurons (as shown in Fig. 6).

### Synchrony receptive fields in auditory and visual modalities

**Figure 12:**
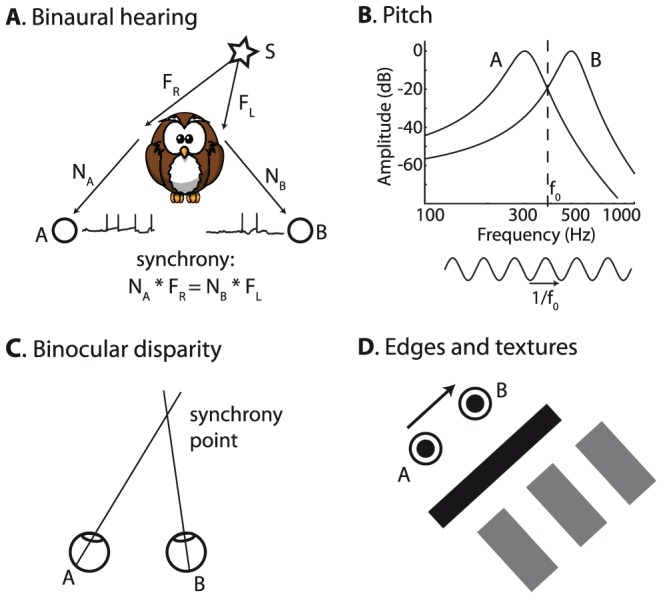
Synchrony receptive fields in the auditory and visual modalities. A, Binaural hearing with realistic sound diffraction. The sound S arrives at the two ears as a binaural signal (F_R_*S, F_L_*S), where F_R_ and F_L_ are location-dependent filters, and is subsequently processed by two monaural neurons with receptive fields N_A_ and N_B_. The synchrony receptive field is the set of source locations such that N_A_ * F_R_ = N_B_ * F_L_. B, Pitch. Two monaural neurons with different preferred frequencies fire in synchrony for a pure tone or resolved partial with frequency 1/f0, at the intersection of the two amplitude spectra (provided that the phase difference is compensated by appropriate delays). C, Binocular disparity. Two retinal ganglion cells fire in synchrony when there is an object at the convergence point of their fixation lines. D, Edges and textures. Two visual neurons with circular receptive fields fire in synchrony to images that are invariant to translations of the vector linking the two receptive field centers: edges with the same orientation and spatially periodic textures with the period given by that vector.

Finally, I will show how the concepts I have exposed apply to a few auditory and visual examples (Fig. 12). In Fig. 7A, I illustrated the notion of structured stimulus in a simplified description of binaural hearing, where the sound arrives at the two ears with an interaural delay that depends on the source direction. In reality, bina ural cues are more complex because the sound is diffracted by the head and pinnae, and even the body (Fig. 12A). The correct physical description is that the two monaural signals are two linearly filtered versions of the original signal S: S_L_ = F_L_*S, S_R_ = F_R_*S (* is the convolution). These location-specific filters are called head-related impulse responses and are more complex than pure delays (in particular, ITD is frequency-dependent [67]). I consider two monaural neurons A and B on opposite sides with different receptive fields N_A_ and N_B_. These neural filters represent basilar membrane filtering around a characteristic frequency (CF), and include an outgoing axonal delay. Thus, they may differ both in CF and in axonal delay. In the framework I have described, these two neurons have synchronous responses when N_A_*F_L_*S = N_B_*F_R_*S, that is, their SRF includes all acoustical filter pairs (F_L_, F_R_) such that N_A_*F_L_ = N_B_*F_R_, meaning that the combination of neural and acoustical filtering match on both sides. Therefore this is a spatial field, and synchrony signals source location independently of source signal. A spiking neural model based on these properties can accurately estimate the location of previously unheard sounds in a realistic virtual acoustic environment [68]. This corresponds to the idea that the tuning properties of binaural neurons may come not only from mismatches in axonal delay but also in the preferred frequency of their monaural inputs [69]–[71]. A prediction from this theory is that the preferred ITD of a binaural neuron can depend on sound frequency, because ITDs depend on frequency when diffraction is taken into account [67]. This property has indeed been observed in binaural neurons of many species [72], [73]. More specifically, the theory predicts that the frequency-dependence of preferred ITDs should match the corresponding quantities in the acoustical filters, which can be measured.

We may also look at the SRF of two monaural neurons on the same side (Fig. 12B). In Fig. 7B, I only considered neurons with identical auditory filters but different delays. If the two neurons have different filters N_A_ and N_B_, then synchrony occurs when N_A_*S = N_B_*S (S is the sound). Looking at this identity in the frequency domain, this means that the two filters must agree at all frequencies where the sound S has power. The synchrony identity means that both the phase and the amplitude spectrum of the filters must agree at all frequency components of S. If the two neurons have different CFs, then this can only occur at the frequency f_0_ where the two amplitude spectra agree. In addition, the phases agree if the difference in delays exactly compensates the difference in phase delays of the filters. Therefore, the SRF is a pure tone with frequency f_0_ - or a resolved partial harmonic of a complex sound (that is, only one frequency component falls in the bandwidth of filters N_A_ and N_B_). In summary, only one specific type of sound elicits patterns of synchrony in monaural neurons: periodic sounds, which are associated with pitch in humans. This produces a new theory of pitch perception, generalizing temporal models, according to which pitch is represented by the pattern of synchrony across frequency (CF) and time (axonal delay). It offers a solution to the two major problems of temporal models of pitch: 1) that they require large axonal delays (as large as the maximum period of a pitch-evoking sound, about 30 ms), 2) that they do not distinguish resolved and unresolved harmonics, while there is a perceptual difference between these two types of pitch-evoking sounds [74]. In the synchrony pattern hypothesis, large axonal delays are not necessary, because mismatches in CF can play this role, and resolved harmonics produce wider synchrony patterns, as they also include neurons with different CFs.

The analog of binaural hearing in vision is binocular disparity (Fig. 12C). Consider two retinal ganglion cells in different eyes, which move with microsaccades (tremor). The two cells fire in synchrony when they see the same dynamic stimulus through their receptive field (retinal ganglion cells are known to fire with millisecond precision [5], [75], [76]). This occurs when there is an object at the convergence point of their fixation lines (connecting their retinal position to the pupil). Thus the synchrony receptive field is a three-dimensional spatial receptive field. Synchrony patterns across the two eyes reflect the structure of the binocular stimulus, which comes from the fact that a single object produces the two retinal images. The hypothesis that depth perception is mediated by the detection of synchrony between two retinal ganglion cells (presumably by a neuron in V1) predicts that decorrelating the images in the two eyes should disrupt depth perception.

In a similar way, the SRF of two monocular visual neurons with circular receptive fields (e.g. neurons in the lateral geniculate nucleus of the thalamus) is the set of images that are unchanged by translations of the vector connecting the centers of the two receptive fields (Fig. 12D). These are edges with the same orientation and spatially periodic textures with the period given by that vector.

## Discussion

To understand the functional role of synchrony, I introduced the concept of *synchrony receptive field:* the set of stimuli that produce synchronous responses in a given neuron pair or group. In a heterogeneous population of neurons, synchrony reflects the structure of stimuli, for example a constant activation ratio between two olfactory receptors responding to an odor. This structure can then be detected by postsynaptic neurons which are sensitive to synchrony. This framework applies to many perceptual tasks, such as recognizing an odor or locating a sound source. I will first comment these results from a computational perspective, and then discuss the biological plausibility of this proposition.

Over the last century, the operating function of neurons has been mainly described in terms of firing rates, and this point of view has led to important developments in computing, from the perceptron [77] to modern artificial neural network theories for pattern recognition [78]. More recently, experimental evidence and theoretical studies, showing the importance of the temporal coordination of spikes [5], [13], have triggered considerable interest for spiking neuron models in computational neuroscience [79]. However, few theories of computation are specifically spike-based, as I have proposed here. Fig. 5B illustrates a fundamental difference between synchrony-based computation and traditional neural network theory: a formal neuron (e.g. perceptron) fires when the stimulus is on one side of a hyperplane, while two neurons fire in synchrony when the stimulus is close to a hyperplane.

In this framework, synchrony in neurons with heterogeneous receptive fields reflects some structure in the stimulus (for example, the periodicity of a pitch-evoking sound). The computational interest stems from the fact that structure is invariant to many aspects of the stimulus: for example, receptor coverage ratios are invariant to odor concentration in a turbulent odor plume, and binaural cues in sound localization are invariant to the signal produced by the source. This computational principle applies to many perceptual tasks in all sensory modalities, and it may also apply to the exploration of sensorimotor contingencies [80]. Robustness to noise stems from the fact that incoherent signals result in an absence of response (no synchrony) rather than in a false response. This relates to the idea that meaningful structure in an image (e.g. edges) is what could not occur by chance in a random image, a principle called “Helmholtz principle” that was recently successfully applied in computer vision [81].

At behavioral level, invariance is a striking aspect of perception: translation invariance in vision [82], concentration invariance in olfaction [65], acoustic scale invariance in hearing [83]. On the other hand, neural responses often vary with many aspects of sensory stimuli. This theory agrees with these observations because the spatial structure of synchrony is invariant but individual neural responses are variable. It relies on two main assumptions: that neurons can synchronize to a similar signal, and that postsynaptic neurons can detect this synchrony. Both properties are seen when neurons are fluctuation-driven (rather than mean-driven), which is in agreement with the temporal irregularity of spike trains *in vivo* [84] and with direct intracellular measurements [40]. Spike timing reproducibility has been observed *in vivo* in early sensory areas [5], [58], but also more recently in the sensory cortices, although at longer timescales [85], [86]. The sensory examples I have chosen are all thought to be processed in subcortical areas, at least for the neurons for which SRFs are defined: odor recognition in the olfactory bulb, sound localization and pitch perception in the auditory brainstem, binocular disparity in the retina and thalamic relay cells (with coincidence detection in the primary visual cortex). There is stronger evidence for the reproducibility of spike timing in these subcortical areas. However, for this theory, stimulus-locked reproducibility is a sufficient but not necessary condition: as I previously remarked (Fig. 6), there may be stimulus-specific synchrony without trial-to-trial reproducibility, if there is a shared source of variability (e.g. activity of the local network, or feedback from other areas). Finally, I have shown that the neural circuits that detect structure-specific synchrony can spontaneously emerge under the effect of spike-timing-dependent plasticity. This was expected because modeling studies have shown that STDP tends to select correlated inputs [20], [21].

In many theories of spiking neural networks (with the notable exception of liquid state machines [23]), neural heterogeneity is seen as a source of noise to be averaged out: the unit of computation is a neural population or “neural mass” [22]. On the contrary, in this theory, it is specifically because of neural heterogeneity that synchrony carries meaningful information. This has some interest for neuroengineering. Indeed, a major problem in low-consumption neuromorphic circuits is that there is substantial variability in neuron properties, which makes it difficult to specify a precise neuron model [87]. In the framework I have described, this variability can be exploited.

How can this theory be experimentally tested? I have mentioned a few predictions in the specific cases of ITD processing and binocular disparity. More generally, a straightforward approach is to measure synchrony receptive fields, by examining how the cross-correlogram of a given neuron pair varies with stimuli, and in particular with the structure of the stimulus, using multielectrode recordings. Previous studies in the olfactory and visual systems support the idea of stimulus-specific synchrony [88], [89], but new experiments should specifically test whether synchrony is related to the structure of the stimulus, for example whether it is robust to changes in intensity.

## Methods

All neuron models were simulated with the Brian simulator [90].

### Models of duration selectivity (Fig. 1–4)

Neurons with rebound spiking are modeled with the following membrane equation:
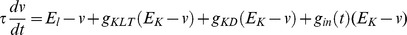
where v is the membrane potential, τ is the membrane time constant (20 ms in Fig. 1, random between 10 and 50 ms in Fig. 2–4), g_KLT_ is the low-threshold K+ conductance (in units of the leak conductance), g_KD_ is the delayed-rectifier K+ conductance, g_in_(t) is the inhibitory synaptic conductance, E_l_ = −35 mV is the leak reversal potential and E_K_ = −90 mV is the K+ reversal potential (note that the resting potential is smaller than E_l_ because of the low-threshold K+ conductance). The low-threshold K+ conductance depends on voltage through the following equation:
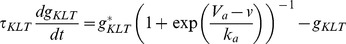
where τ_KLT_ is the time constant (100 and 400 ms for neurons A and B in Fig. 1, random between 300 and 500 ms in Fig. 2), V_a_ = −70 mV is the half-activation voltage, k_a_ = 5 mV is the activation Boltzmann factor and g_KLT_* is the maximal conductance (1 in Fig. 1, random between 1 and 1.4 in Fig. 2–4). Thus this hyperpolarizing conductance increases with voltage. A spike is produced when v reaches v_t_ = −55 mV, then the membrane potential is reset to −70 mV and the delayed-rectifier K+ conductance is set to g_KD_ = 2. This conductance then decays exponentially:
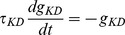
where τ_KD_ = 300 ms. This prevents the neuron from producing bursts of spikes. Synaptic conductances are pulses of amplitude g_in_ = 5 (in units of the leak conductance) and variable duration. This choice of parameter values is explained in Text S1.

Coincidence detectors are modeled as noisy integrate-and-fire models:
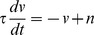


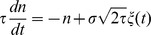
where τ = 5 ms is the membrane time constant, n(t) is a filtered noise with standard deviation σ = 0.2 (ξ(t) is white noise). The resulting standard deviation of the membrane potential v is 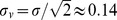. In Fig. 2, each presynaptic spike increases v by an amount 1/N, where N is the number of presynaptic neurons, and a spike is produced when v = 1. The 1/N scaling factor ensures that the postsynaptic neuron fires with probability 1/2 when inputs are synchronous. After spiking, the membrane potential is reset to 0.

In Fig. 3B, inputs are modeled as synaptic conductances rather than currents, i.e.,
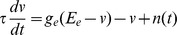


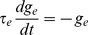
where τ_e_ = 2 ms is the excitatory time constant and E_e_ = 4.7 is the excitatory reversal potential (relative to the threshold; this corresponds to E_e_ = 0 mV for a threshold v_t_ = −55 mV and E_l_ = −70 mV). Each presynaptic spike increases g_e_ by an amount α/N, where α is calculated so that the PSP produced by a conductance increase of size α reaches the spike threshold v_t_ = 1, with the approximation that the synaptic driving force is E_e_−1/2 (1/2 being the average of the resting potential 0 and the spike threshold 1). This gives the following formula:
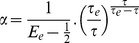
In Fig. 3C, the coincidence detectors are described by the same equations as the model with rebound spiking, with τ_KLT_ = 400 ms, g_KLT_* = 2.1, τ = 10 ms, and inputs are also modeled as synaptic conductances, with E_e_ = 0 mV. The noise is scaled so as to represent the same proportion of the difference between resting potential and threshold (which gives 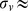
1 mV). Each presynaptic spike increases g_e_ by an amount α/N, where α is calculated as above, but taking into account the total conductance of the cell at rest (leak plus K+) and the resting potential (empirically determined as 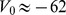 mV). The resulting formula is:
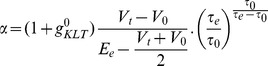
where 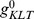 is the K+ conductance at rest (calculated from the activation curve) and 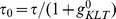.

### Learning models (Fig. 4–5, 11)

Synaptic weights w evolve with homeostasis and spike-timing-dependent plasticity (STDP). Homeostasis is defined by:
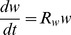


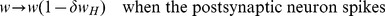
STDP is defined by a modification of synaptic weight that depends on the timing of pre- and postsynaptic spikes:
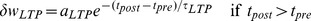
The time constant is 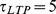 ms for the duration model and 3 ms for the olfaction model. In Fig. 4, 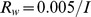 (where I is the duration of a stimulus presentation), 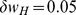 and 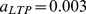. In Fig. 11, 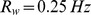, 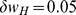 and 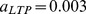.

### Olfactory models (Fig. 8–11)

The fluctuations of concentration in an odor plume are described by a half-wave rectified Ornstein-Uhlenbeck process:
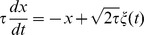
with time constant τ = 75 ms [63], where the odor concentration is proportional to [x]^+^ ( = max(x,0)). Each of the N = 5000 olfactory receptor neurons has an odor-specific affinity which depends on its type, and a global sensitivity, which is neuron-specific. Thus, each odor can be represented as an N-dimensional vector of binding coefficients b_i_, combining affinity and sensitivity (b_i_ = a_i_.s_i_). To generate an odor, we draw random binding coefficients logarithmically distributed between 10^−3^ and 10^3^. The transduction current of a receptor cell is a Hill function of odor concentration:
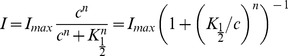
where c is the (time-varying) concentration, n = 3 is the Hill coefficient, related to the slope of the curve [64], I_max_ is calculated to produce a maximum firing rate of 40 Hz, and K_1/2_ is the half-activation concentration, which is the inverse of the binding coefficient: K_1/2_ = 1/b_i_. The concentration varies in time as c(t) = c_0_.x(t) (x(t) are the random fluctuations defined above). Note that this latter parameter depends on both the neuron and the odor. Currents are transformed into spike trains through an integrate-and-fire model:
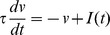
where τ = 20 ms is the membrane time constant (except Fig. 7C, where it is uniform between 15 and 25 ms), and I(t) is the transduction current. A spike is produced when v = 1, then the membrane potential is reset to 0.

Coincidence detectors are defined as for models of duration selectivity, with τ = 8 ms and σ = 0.15. In Fig. 6, 400 such postsynaptic neurons are split in two groups tuned to either odor A or B. Each postsynaptic neuron receives excitatory synapses from presynaptic neurons with similar binding coefficients for the target odor. Specifically, the range of binding coefficients is divided in 200 equal layers (in logarithmic scale), and each layer is associated with one postsynaptic neuron, which receives inputs from all receptors with binding coefficients in that layer. The synaptic weight is 1/n, where n is the number of presynaptic neurons (12.4±3.6). In Fig. 10C (new wiring), odd index neurons only receive inputs from receptors with τ<20 ms and even index neurons from those with τ>20 ms. We compensate by doubling the size of layers for binding coefficients, so that the average number of presynaptic neurons is unchanged.

### Measures of precision and reliability

Precision and reliability measures (Fig. 6) are obtained from shuffled auto-correlograms (SAC) [14] - the average cross-correlogram between distinct trials. These are normalized by 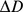, where 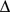 is the time bin and D is the duration of trials. After removing the baseline (equal to r^2^, where r is the firing rate), the precision is defined as the half-width of the SAC, and the reliability as the normalized integral of the peak:
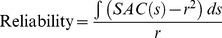
which gives a number between 0 and 1, where 0 is obtained for independent spike trains and 1 when comparing a spike train with a jittered copy (i.e., perfect synchrony if the timescale is 0 ms). This corresponds to the *total correlation* coefficient in [91].

### Shared variability

In Fig. 7, stimuli, shared input and private noise are generated as Ornstein-Uhlenbeck processes with time constant 10 ms. Stimuli and shared inputs have the same standard deviation and that of the private noise is set by the signal-to-noise ratio. Neurons are modeled as integrate-and-fire units:
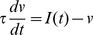
with τ = 10 ms and I(t) is the total input. The spike threshold is 1 and the reset is 0. Shuffled and cross-correlograms are calculated as in Fig. 6 (previous paragraph), averaged over many trials.

## Supporting Information

Text S1 Supplementary methods. This supplementary text describes the properties of the duration model, in relationship with the parameter values.
(PDF)
