# Supplementary material for: Computing with Neural Synchrony
Source: PLoS Comput Biol. 2012 Jun 14;8(6):e1002561. doi: 10.1371/journal.pcbi.1002561 (PMC3375225; doi:10.1371/journal.pcbi.1002561)
Supplement: Text S1 — Supplementary methods. This supplementary text describes the properties of the duration model, in relationship with the parameter values. (PDF) [file pcbi.1002561.s001.pdf]

Computing with neural synchrony

Romain Brette (romain.brette@ens.fr)
1. Laboratoire Psychologie de la Perception, CNRS and Université Paris Descartes, Paris, France
2. Département d'Etudes Cognitives, Ecole Normale Supérieure, Paris, France

Text S1 -Supplementary Methods

Synchrony receptive fields in the duration selectivity model

In the duration selectivity model, the neuron models with post-inhibitory rebound are defined as follows:

$$\tau \frac{dv}{dt} = E_l - v + g_{max} g_K (E_K - v)$$

$$\tau_K \frac{dg_K}{dt} = \left(1 + e^{\frac{V_a - v}{k_a}}\right)^{-1} - g_K$$

where I neglected inhibitory and delayed rectifier conductances because they have no role in the calculations below. We set V_a = -70 mV, k_a = 5 mV, E_K = -90 mV and E_l = -35 mV (so that the neuron spikes when the K+ channels are closed). The spike threshold is v_t = -55 mV, and the neuron is reset to v_r = -70 mV after a spike. Conductances are in units of the leak conductance.

<u>1. Resting condition</u>

The resting potential is:

$$v_0 = \frac{E_l + g_{max}\left(1 + e^{\frac{V_a - v_0}{k_a}}\right)^{-1} E_K}{1 + g_{max}\left(1 + e^{\frac{V_a - v_0}{k_a}}\right)^{-1}}$$

This must be smaller than the spike threshold v_t, which means:

$$E_l - v_t + g_{max}\left(1 + e^{\frac{V_a - v_0}{k_a}}\right)^{-1} (E_K - v_t) < 0$$

which is a condition on g_{max}:

$$g_{max} > \frac{E_l - v_t}{v_t - E_K}\left(1 + e^{\frac{V_a - v_0}{k_a}}\right)$$

The limit condition is when $v_0 = v_t$, therefore the general condition on g_{max} is:

$$g_{max} > \frac{E_l - v_t}{v_t - E_K}\left(1 + e^{\frac{V_a - v_t}{k_a}}\right)$$

With the values defined above, this means:

$$g_{max} > 0.6$$

2. K+ conductance at stimulus offset

When the neuron is strongly inhibited, v=E_K. So the K conductance decays exponentially to the equilibrium value, which is almost 0. Therefore:

$$g_K(t) = g_K(0)e^{-t/\tau_K}$$

where t is the duration of inhibition.

3. Minimum inhibition duration

The neuron spikes when the inhibitory stimulus is long enough, that is, when the K conductance is such that the equilibrium potential at stimulus offset is above threshold:

$$\frac{E_l + g_K E_K}{1 + g_K} > V_t$$

which means:

$$g_K < \frac{E_l - V_t}{V_t - E_K}$$

Using the formula obtained in 1.2:

$$t > \tau_K \log g_K(0) + \tau_K \log \frac{v_t - E_K}{E_l - v_t}$$

where t is the stimulus duration (note that the resting condition implies that this is a positive number).

4. Rebound spike latency

The latency decreases with increasing inhibition. For long inhibition, the K channels are closed, therefore the minimum spike latency is:

$$t_{min} = \tau \log \frac{E_l - E_K}{E_l - v_t} \approx \tau$$

assuming that activation of K+ channels is slow (compared to t_{min}). The maximum latency (for the minimum stimulus duration) is +∞. The spike latency for an arbitrary duration can be calculated, but is a complicated function of the different parameters. As a result, the synchrony condition (duration for which two neurons with different parameters produce spikes with the same latency) cannot be analytically calculated.

5. Parameter distribution

We use the calculations above to set an appropriate distribution for the following parameters: τ, τ_K, and g_{max} (the other parameters are fixed).

First, the asymptotic spike latency for long durations, approximately equal to τ, should be heterogeneous enough, so that two neurons are generally not synchronous for long durations. In the simulations, τ is drawn uniformly between 10 and 50 ms, which gives a standard deviation of 11.5 ms (the time constant of coincidence detectors is 5 ms).

We then choose the other parameters so that the minimum stimulus duration that elicits a spike is heterogeneous enough, in the range of interest (about 100-600 ms). This minimum is given by the following formula:

$$m = \tau_K \log g_K(0) + \tau_K \log \frac{v_t - E_K}{E_l - v_t}$$

that is:

$$m = \tau_K \log g_{max} + \tau_K \log \frac{v_t - E_K}{E_l - v_t} - \tau_K \log\left(1 + e^{\frac{V_a - v_0}{k_a}}\right)$$

Remember that the resting condition is:

$$g_{max} > \frac{E_l - v_t}{v_t - E_K}\left(1 + e^{\frac{V_a - v_t}{k_a}}\right)$$

This suggests that we express g_{max} as follows:

$$g_{max} = \frac{E_l - v_t}{v_t - E_K} x \approx 0.57x$$

where $x>1.05$. The minimum duration now reads:

$$m = \tau_K \log \frac{x}{1 + e^{\frac{V_a - v_0}{k_a}}}$$

(where v_0 implicitly depends on x). In the simulations, x is drawn uniformly between 1.7 and 2.5 and τ_K is drawn uniformly between 300 and 500 ms. This gives minimum durations with the correct order of magnitude.
